# Exosome-Mediated miR-4792 Transfer Promotes Bladder Cancer Cell Proliferation via Enhanced FOXC1/c-Myc Signaling and Warburg Effect

**DOI:** 10.1155/2022/5680353

**Published:** 2022-01-19

**Authors:** Jian-Hong Wu, Ke-Ning Sun, Zhi-Hao Chen, Yi-Jun He, Lu Sheng

**Affiliations:** Department of Urology, Huadong Hospital Affiliated to Fudan University, Shanghai 200040, China

## Abstract

Bladder cancer is the second-most common malignancy in the urogenital system and the most common in men. However, our understanding of the driving mechanisms of bladder cancer remains incomplete. The forkhead box (FOX) family of transcription factors is implicated in urogenital development and bladder malignancies. Many exosomal microRNAs have been identified as regulators and mediators of the expression of FOX, including the expression of FOXC1. miR-4792 has been known as a tumor miRNA suppressor. However, the function of miR-4792/FOXC1 signaling in bladder cancer development remains unknown. Here, we studied the role of miR-4792/FOXC1 signaling in bladder cancer by using multiple bladder cancer cell lines and bladder cancer mouse models through *in vitro* and *in vivo* approaches. We showed that FOXC1 is highly expressed in multiple bladder cancer cell lines and bladder tumor tissues. The knockdown of FOXC1 expression in bladder cancer cell lines decreases c-Myc expression levels, retards cell growth, and reduces aerobic glycolysis (also known as the Warburg effect) and lactic acid content. By contrast, the overexpression of FOXC1 elicits the opposite effects. FOXC1-downregulated bladder cancer cells form significantly smaller tumors *in vivo*. The inhibition of c-Myc reverses the effects of FOXC1 overexpression and leads to reduced cell proliferation, aerobic glycolysis, and lactic acid content. miR-4792 expression is downregulated in bladder tumor tissues. miR-4792 exposure to bladder cancer cells reduces the expression levels of FOXC1 and c-Myc, slows down cell growth, and decreases aerobic glycolysis and lactic acid content. However, the enhanced miR-4792 expression elicits opposite effects. These findings provided the first evidence that the exosome-mediated delivery of miR-4792 could play an important role in bladder cancer development through the downregulation of FOXC1 and c-Myc, which further inhibited aerobic glycolysis and lactic acid content.

## 1. Introduction

Bladder cancer is the second-most common malignancy in the urogenital system [1]. Urothelial cell carcinoma, which originates from urothelial cells, accounts for more than 90% of all bladder cancer cases [2]. The highest risk factors of bladder cancer are smoking, being a male, and age. The prevalence of bladder cancer will increase significantly in the next two decades because of population aging [3].

Forkhead box (FOX) proteins are composed of a set of evolutionarily conserved transcription factors, which share a common DNA binding structure named the FOX structure [4]. FOX family proteins are involved in many biological processes, especially cell apoptosis and differentiation [5]. FOXC1 was initially identified to have a critical role in Axenfeld–Rieger syndrome [6]. However, in recent years, increasing evidence has shown that the deficiency of FOX proteins is implicated in tumorigenesis [7]. Multiple studies have indicated that FOXC1 upregulation is related to poor prognosis in many cancer types, including acute myeloid leukemia [8], basal-like breast cancer [9], liver cancer [10], pancreatic ductal adenocarcinoma [11], and gastric cancer [12].

MicroRNAs (miRNAs) are noncoding RNAs that can regulate the gene expression of many targets mostly at a post-transcriptional level and participate in almost all biological processes, including tumorigenesis [13]. Cell-free miRNAs are unstable; however, exosomes, which contain multiple miRNAs, can protect them from being degraded and deliver miRNAs over long distances [14]. Indeed, previous studies demonstrated that exosome-mediated mRNA or miRNA transfer plays an important role in cell communication in normal development and diseases, including malignancies [15]. As a tumor suppressor miRNA, miR-4792 has been shown to be downregulated in multiple cancer types, including uterine leiomyoma, breast cancer, and nasopharyngeal tumor [16–19]. Notably, in nasopharyngeal tumors, miR-4792 inhibits the epithelial-mesenchymal transition, invasion, and metastasis by targeting FOXC1 [16]. It is significantly downregulated in bladder carcinoma [20]. FOXC1 has an important role in oncogenesis and is linked to poor prognosis in several cancer types. However, the role of the miR-4792/FOXC1 axis in bladder cancer development is unknown.

In this study, we determined the expression of miR-4792 and FOXC1 and explored how their loss or overexpression would affect bladder cancer development by using multiple bladder cancer cell lines and bladder cancer tumor models. We also explored how they could affect aerobic glycolysis, which is a hallmark of a majority of cancer cells.

## 2. Methods

### 2.1. Cell Culture

Normal bladder epithelial cells (SV-HUC-1) and bladder cancer cell lines (253J, 5637, J82, SCABER, and T24) were cultured in RPMI-1640 medium (SH30809.01 B, HyClone, Logan, UT, US) and basal medium Eagle (BME; 21010-046, Gibco, Carlsbad, CA, USA) supplemented with 10% fetal bovine serum (FBS; 16000-044, GIBCO) and 1% penicillin-streptomycin (P1400, Solarbio, Beijing, China). All cell lines were maintained in a humidified incubator with 5% CO_2_ at 37°C.

### 2.2. Plasmid Construction

The coding sequence of FOXC1 (NM_001453.3) was amplified with primers containing Hind III and EcoR I restriction enzyme cutting sites. The recovered and purified sequences were integrated into the pCDNA3.1(+) vector, which contains the neogene as a selection marker. The primers used for the plasmid construction are as follows:

  FOXC1-F: 5ʹ-CCCAAGCTTATGCAGGCGCGCTACTC-3ʹ (Hind III)
  FOXC1-R: 5ʹ-CGGAATTCTCAAAACTTGCTACAGTCGTAGAC-3ʹ (EcoR I)

shRNAs targeting FOXC1 were cloned into the pLKO.1-puro plasmid, which contains a puromycin selection marker. shRNAs used for plasmid construction are listed as follows:

  FOXC1 site 1 (590-608): 5ʹ-GCGGCGAGCAGAGCTACTA-3ʹ
  FOXC1 site 2 (1004-1022): 5ʹ-CCTACAACATGTTCGAGAA-3ʹ
  FOXC1 site 3 (2164-2182): 5ʹ-GCTTTCGTCTACGACTGTA-3ʹ

### 2.3. Cell Transfection and Establishment of Stable Cell Lines

In the logarithmic growth phase (usually after several passages since the recovery from cryopreservation), bladder cancer cell suspension was prepared and diluted to 1 × 10^6^ cells/ml. An aliquot of 2 ml of cell suspension was seeded in each well of six-well plates and maintained overnight at 37°C in a 5% CO_2_ incubator. When the cells reached a confluency of 60%–70%, the cells were transfected with an empty vector (pLKO.1-puro), shNC (negative control), shFOXC1-1, shFOXC1-2, and shFOXC1-3 plasmids; with an empty vector (pCDNA3.1(+)) and oeFOXC1 plasmid; or with miR-NC, miR-4792 inhibitor (antagomir, a small synthetic RNA), and miR-4792 mimic (agomir, chemically-modified double-strand miRNA) by using Lipofectamine 2000. The medium was removed 24 h after transfection, and a fresh complete medium was added to the wells for another 48 h of culture. For the FOXC1 knockdown, the transfected cells were then split and added into a new medium containing Geneticin (G418) for the selection of neomycin-resistant cells. The cells were split every 3-4 days until Geneticin (G418)-resistant clones were formed. The clones were selected and expanded in 96-well plates. For the FOXC1 overexpression, puromycin was used for selection. The procedures for puromycin-resistant cells were similar to the procedures for Geneticin selection.

The sequences of the miR-4792 mimic and miR-4792 inhibitor are listed as follows:

  hsa-miR-4792 mimic (agomir): 5ʹ-CGGUGAGCGCUCGCUGGC-3ʹ
  hsa-miR-4792 inhibitor (antagomir): 5ʹ-GCCAGCGAGCGCUCACCG-3ʹ
  miR-NC: 5ʹ-CAGUACUUUUGUGUAGUACAA-3ʹ

### 2.4. Isolation and Identification of Human Adipose-Derived Mesenchymal Stem Cell (hAMSC) Exosomes

miR-4792 is the most abundant in adipose-derived mesenchymal stem cells (AMSCs) [21]; as such, exosomes were extracted from hAMSC exosomes (hAMSC_exo) through differential centrifugation at 10,000 × g and 4°C for 30 min. The supernatants were then supplemented with 1 × PBS, transferred to 5 mL ultrahigh-speed centrifugal tubes, and centrifuged twice at 17,000 × g and 4°C for 2 h. The supernatants were collected and centrifuged again by repeating the abovementioned procedures. The pellets were resuspended in 1 × PBS, filtered with a 0.22 *μ*m filter, and stored at −80°C until further analysis. The extracted exosomes (hAMSC_exo1 and hAMSC_exo2) were identified by western blot with exosomal markers (TSG101 and CD81). Anti-TSG101 (ab125011) and anti-CD81 (ab109201) antibodies were purchased from Abcam.

### 2.5. Exosome Tracing by PHK-67 Fluorescence Staining

PKH-67 exosome green fluorescent dye (UR52303, Umibio) was used to trace the exosomes that were endocytosed by bladder cancer cells. A PKH-67 working solution was prepared in accordance with the manufacturer's instructions. In brief, a “PKH-67 linker” was mixed with “Diluent C” at a ratio of 1 : 9 in the dark at room temperature. The PKH-67 staining working solution was added to the exosome solution to stain the exosomes. After they were thoroughly mixed, the mixture was incubated at room temperature in the dark for 10 min. A fluorescence microscope (BX51, OLYMPUS) was used to monitor fluorescence-stained cells.

### 2.6. Quantitative Real-Time PCR

The relative mRNA levels in different cell groups were assessed through quantitative real-time PCR (qRT-PCR). In brief, RNA was extracted with TRIzol (1596-026, Invitrogen; Carlsbad, CA, USA), reverse transcribed to cDNA by using a RevertAid first-strand cDNA synthesis kit (Fermentas, Hanover, MD, USA), and further amplified using SYBR Green qPCR Master mixes (#K0223, Thermo Fisher, Rockford, IL, USA) in accordance with the manufacturer's instructions. The relative mRNA levels were calculated with the 2^−ΔΔCt^ method after they were normalized to the reference gene (GAPDH). The primers used for qRT-PCR are listed as follows:

*Homo sapiens* forkhead box C1 (FOXC1) and mRNA (NM_001453.3)
    Primer F 5ʹ-CATAGCCAGGGCTTCAGCG-3ʹ
    Primer R 5ʹ-TGCAGGTTGCAGTGGTAGGTC-3ʹ
*H. sapiens* glyceraldehyde-3-phosphate dehydrogenase (GAPDH), transcript variant 2, and mRNA (NM_001256799.2)
    Primer F 5ʹ-AATCCCATCACCATCTTC-3ʹ
    Primer R 5ʹ-AGGCTGTTGTCATACTTC-3ʹ
hsa-miR-4792 (MIMAT0019964)
    RT-Primer
    5ʹ-GTCGTATCCAGTGCAGGGTCCGAGGTATTCGCACTGGATACGACGCCAGC-3ʹ
    PCR Primer
    Primer F 5ʹ-CGCGCGGTGAGCGCTC-3ʹ
    Primer R 5ʹ-AGTGCAGGGTCCGAGGTATT-3ʹ
*H. sapiens* RNA, U6 small nuclear 1 (RNU6-1), and small nuclear RNA (NR_004394.1)
    Primer F 5ʹ-CTCGCTTCGGCAGCACA-3ʹ
    Primer R 5ʹ-AACGCTTCACGAATTTGCGT-3ʹ

### 2.7. Western Blot Analysis

Cells were lysed using RIPA tissue cell rapid lysate (P0013, Beyotime). The lysates were separated through electrophoresis on SDS-PAGE gels. Proteins were transferred to polyvinylidene fluoride membranes and blocked with 5% nonfat milk. The membranes were first incubated with primary antibodies at predetermined concentrations and then with secondary antibodies. Protein levels were assessed using a chemiluminescent imaging system (Tanon 5200, Shanghai, China). Anti-FOXC1 (ab223850), anti-c-Myc (ab32072), anti-TSG101 (ab125011), and anti-CD81 (ab109201) antibodies were purchased from Abcam. The anti-GAPDH (#5174) antibody was purchased from CST.

### 2.8. Cell Counting Kit-8 (CCK-8) Assay

The CCK-8 assay was conducted using a cell proliferation and cytotoxicity assay kit (SAB, CP002; College Park, MD, USA). In brief, 100 *μ*L of cell suspension with 2 × 10^3^ bladder cancer cells (253J, 5637, or T24 cell lines) was added to each well of a 96-well plate for overnight culture in an incubator. Then, the cells were divided into groups of different treatments. After the treatment and at specific time points, 10 *µ*L of CCK-8 solution was added to each well. Cell viability was estimated by measuring the absorbance at 450 nm with a microplate reader.

### 2.9. Cell Mito Stress Test

The oxygen consumption rate (OCR), which measures the cellular respiration rate, is an important indicator of mitochondrial function. OCR was measured using a Seahorse XF24 analyzer (Seahorse Bioscience, Billerica, MA, USA). After the cells were incubated for 48 h, the growth medium from each well was removed, leaving a small amount (approximately 50 *µ*L) of the medium. The cells were rinsed twice using 1,000 *µ*l of a prewarmed assay medium (XF base medium containing 1 mM sodium pyruvate, 2 mM glutamine, and 25 mM glucose; pH 7.4), and approximately 50 *µ*L of medium was left in each well. A total of 475 *µ*L of the assay medium was added to reach a final volume of about 525 *µ*L in each well.

The cells were subsequently incubated at 37°C without CO_2_ for 60 min to allow the cells to be equilibrated with the assay medium. The prewarmed mixture of antimycin A, rotenone, FCCP, and oligomycin was loaded into injector ports A, B, and C of a sensor cartridge at specific time points. The OCR was measured under the basal condition, and 0.5 *µ*M oligomycin, 1 *µ*M FCCP, 1 *µ*M rotenone, and antimycin A mixture were sequentially added at specific time points.

### 2.10. Glycolysis Stress Test Assay

The extracellular acidification rate (ECAR), which measures extracellular lactic acid, is an indicator of the strength of aerobic glycolysis. ECAR was measured using a Seahorse XF24 analyzer (Seahorse Bioscience, Billerica, MA, USA). After the cells were incubated for 48 h, the medium was replaced with an assay medium (XF base medium DMEM containing 2 mM glutamine). The cells were incubated at 37°C without CO_2_ for 60 min to allow the cells to be equilibrated with the assay medium. Glucose, oligomycin, and 2-deoxy-D-glucose (2-DG) were diluted in the assay medium to final concentrations of 10 mM, 0.5 *µ*M, and 100 mM, respectively. They were then loaded into ports A, B, and C at specific time points, respectively. A glycolytic stress test assay was conducted in accordance with the manufacturer's instructions (Seahorse Bioscience, Billerica, MA, USA). The ECAR was measured under the basal condition, and 100 mM of 2-DG, 0.5 of *µ*M oligomycin, and 10 mM of glucose were sequentially added.

### 2.11. Biochemical Detection

After the treatment, the cells were collected, and the supernatant was obtained. The lactic acid content was detected using a lactic acid content detection kit (A019-2, Nanjing Jiancheng Bioengineering Institute) and measured via a microassay in accordance with the manufacturer's instructions.

### 2.12. Xenograft Study

A total of 5637 cells (6 × 10^6^) that stably expressed shFOXC1 or shNC (the established stable 5637 cell lines) were subcutaneously injected into the flank region of 4-to-5-week-old male nude mice (Shanghai Laboratory Animal Company, Shanghai, China). The tumor volumes were measured every 3 days, and the mice were sacrificed 33 days after cell implantation. Tumor volumes were calculated using the following equation: volume = 0.5 × *L* × *W* × W, where *L* is the length (the longest diameter), and W is the width (the diameter perpendicular to the length). Tumor xenografts were collected, photographed, weighed, sectioned, and embedded in paraffin for further experiments. Laboratory animal care and experiments were conducted in accordance with the guidelines of the animal ethics and approved protocols of Huadong Hospital Affiliated to Fudan University (Shanghai, China).

### 2.13. Immunofluorescence (IF) Microscopy

Tumor xenografts were fixed with 4% formaldehyde, sectioned to 5–10 *µ*m, and permeabilized with 0.5% Triton X-100. After 30 min of blocking with 1% bovine serum albumin, the sections were incubated with primary anti-Ki67 antibody (ab15580, Abcam; 1 : 1000 dilution) and secondary Alexa Fluor 488-labeled goat anti-rabbit IgG (*H* + *L*) antibody (A0423, Beyotime Biotechnology; 1 : 500 dilution). Afterward, the nuclei were stained and mounted with a hard-set mounting medium containing DAPI (C1002, Beyotime Biotechnology; 1 : 500 dilution). A fluorescence microscope (Leica Microsystems Inc., Buffalo Grove, IL, USA) was employed to examine the stained cells.

### 2.14. Hematoxylin-Eosin Staining

After being fixed and embedded, the tissues were cut into 4–7 *µ*m sections and baked in an oven at 65°C for 30 min. The sections were then dewaxed in xylene (Shanghai Sinopharm) twice for 15 min at a time, immersed in 100%, 95%, 85%, and 75% ethanol for 5 min each, and rinsed with tap water for 10 min. Thereafter, the sections were stained with hematoxylin solution for 5 min, and they were subjected to color separation in ammonia water for a few seconds. After 15 min of rinsing with water, the sections were dehydrated with 70% and 90% alcohol for 10 min, sequentially stained with eosin solution for 1-2 min, and dehydrated with alcohol. After the sections were mounted, their images were captured under a microscope (Eclipse Ni, Nikon) and analyzed using an IMS image analysis system (DS-Ri2, Nikon).

### 2.15. Dual-Luciferase Reporter Gene Assay

The potential binding site between FOXC1 and miR-4792 was predicted, and the corresponding mutant sequence of FOXC1 was constructed into luciferase reporter vectors. The wild-type FOXC1 was also constructed into the vector. The wild-type and mutant FOXC1 vectors and miR-4792 antagomir or agomir were transfected into 293T cells. After 48 h, a dual-luciferase reporter assay kit was used to detect luminescence.

### 2.16. Statistical Analyses

Data were statistically analyzed using GraphPad Prism 7.0 (San Diego, CA, USA). Data were obtained from at least three repetitive experiments and presented as mean ± SD. A one-way ANOVA with Tukey's post hoc tests was applied to compare the means of more than two groups. Data with *p* < 0.05 were regarded statistically significant.

## 3. Results

### 3.1. FOXC1 Is Highly Expressed in Bladder Cancer Cell Lines Compared with That in Normal Bladder Epithelial Cells

FOXC1 is highly expressed in breast, liver, and pancreatic cancers. However, the expression status of FOXC1 in bladder cancer is unknown. In this study, we first checked the expression level of FOXC1 in TCGA bladder cancer (BLCA) and found that the FOXC1 expression in bladder cancer was diverse. In particular, it was highly expressed in some cases, but it was poorly expressed in other cases compared with those in adjacent normal tissues. We further determined the mRNA and protein levels of FOXC1 in several BLCA cell lines through qRT-PCR and western blot. FOXC1 was highly expressed in five BLCA cell lines, namely, 253J, 5637, J82, SCABER, and T24, compared with that in a normal bladder epithelial cell line (SV-HUC-1; Supplementary Figure 1(a)). Notably, FOXC1 was highly expressed in the 253J and 5637 cell lines, poorly expressed in the T24 cell line, and moderately expressed in the J82 and SCABER cell lines (Supplementary Figure 1(a)).

To explore the role of FOXC1 in BLCA cell lines, we established stable FOXC1-knocked down and FOXC1-overexpressing cell lines (Supplementary Figures 1(b)–1(d)). For the knockdown, we used three different shRNAs (shFOXC1-1, shFOXC1-2, and shFOXC1-3) targeting two BLCA cell lines, namely, 253J and 5637, with the highest FOXC1 expression (Supplementary Figures 1(b) and 1(c)). In Figures S1(b) and S1(c), the mRNA and protein levels of FOXC1 were significantly reduced in the knockdown groups (shFOXC1-1, shFOXC1-2, and shFOXC1-3) compared with those of the control or sham (shNC) groups. For the overexpression, we transfected the FOXC1 expression plasmid (oeFOXC1) in T24 cells, which had a relatively lower expression than that of the four other BLCA cell lines (Supplementary Figure 1(d)). In Supplementary Figure 1(d), the mRNA and protein levels of FOXC1 were significantly elevated in this stable cell line. Thus, we established stable BLCA cell lines deficient of or overexpressing FOXC1.

### 3.2. Downregulation of FOXC1 Expression Slows Down *In Vitro* Cell Growth, Decreases OCR and ECAR, Reduces c-Myc Expression, and Inhibits *In Vivo* Tumor Growth

**Figure 1:**
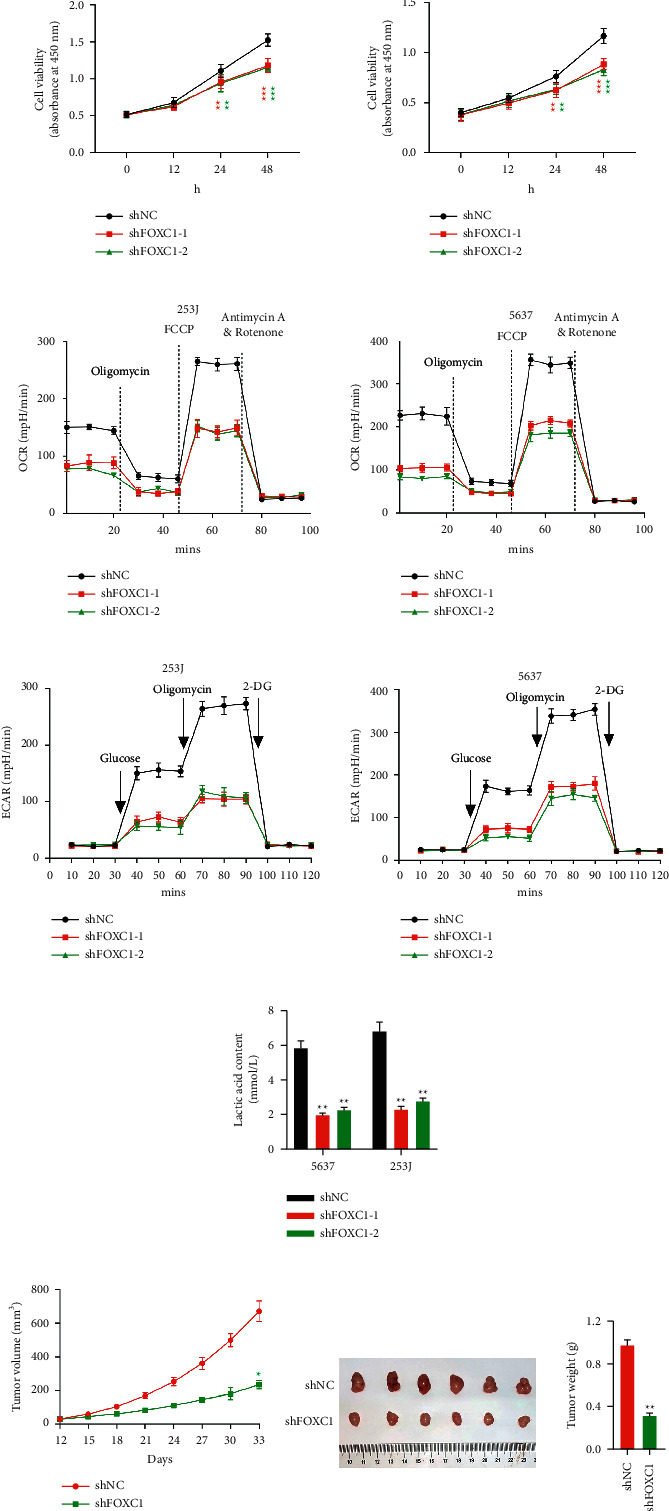
Downregulation of FOXC1 significantly suppressed bladder tumor cell growth and aerobic glycolysis. (a) The CCK8 assay was used to measure the number of 253J and 5637 cells at 0, 12, 24, and 48 h ^*∗∗*^*p* < 0.01, ^*∗∗*^*p* < 0.001 vs. shNC. (b, c) OCR and ECAR were examined using a Seahorse XF24 analyzer. A xenograft nude mouse tumor model was established using 5637 stable cell lines. (d) Lactic acid content was measured. ^*∗∗*^*p* < 0.01 vs. shNC. (e) The tumor size was measured with a caliper. ^*∗*^*p* < 0.05 vs. shNC. shNC: *n* = 6; shFOXC1: *n* = 6. (f) The size of the dissected tumors was indicated by a ruler, and tumor weight was determined. ^*∗∗*^*p* < 0.01 vs. shNC. (shNC: *n* = 6; shFOXC1: *n* = 6).

To explore how FOXC1 affects cell growth in BLCA cells, we measured the cell viability by using a CCK-8 assay in FOXC1-deficient cells. We found that the downregulation of FOXC1 in 253J and 5637 cells significantly reduced their cell viability (Figure 1(a)).

**Figure 2:**
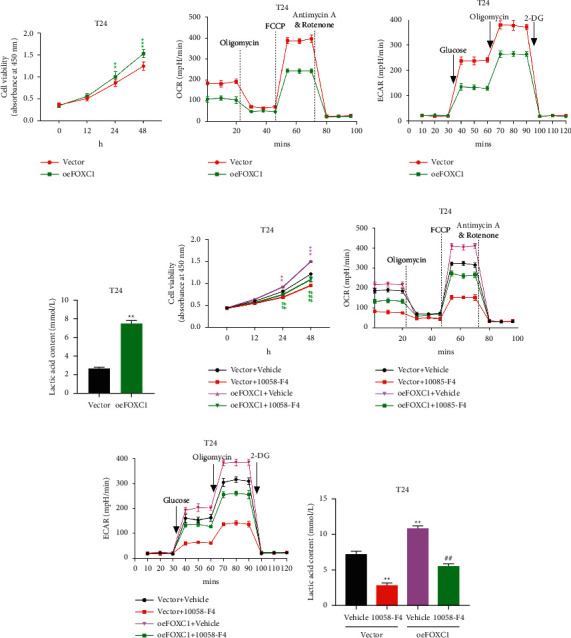
Inhibition of c-Myc significantly reversed FOXC1-induced cell growth and aerobic glycolysis in T24 cell lines. T24 cells were transfected with the FOXC1 plasmid. (a) The CCK8 assay was used to measure the number of cells at 0, 12, 24, and 48 h ^*∗∗*^*p* < 0.01, ^*∗∗∗*^*p* < 0.001 vs. vector. (b, c) OCR and ECAR were measured using a Seahorse XF24 analyzer. (d) Lactic acid content was measured. After 24 h of FOXC1 plasmid transfection, 100 *μ*mol/L of c-Myc inhibitor, namely, 10058-F4, was added to the cells. (e) The CCK8 assay was used to measure the number of cells at 0, 12, 24, and 48 h in different groups. ^*∗∗*^*p* < 0.01, ^*∗∗∗*^*p* < 0.001 vs. vector + vehicle; ##*p* < 0.01, ###*p* < 0.001 vs. oeFOXC1 + vehicle. (f, g) OCR and ECAR were measured using a Seahorse XF24 analyzer in different groups. (h) Lactic acid content was measured. ^*∗∗*^*p* < 0.01 vs. vector + vehicle; ##*p* < 0.01 vs. oeFOXC1 + vehicle.

Aerobic glycolysis is a hallmark of metabolism reprogram in cancers, and cancer cells are marked with increased aerobic glycolysis (also known as the Warburg effect), which might be related to mitochondrial damage. To explore whether FOXC1 promoted BLCA cell proliferation by enhancing aerobic glycolysis, we determined the OCR and ECAR by using a Seahorse XF24 system. In Figure 1(b), at the baseline level, the OCR was significantly lower in the FOXC1 knockdown groups (0–20 min) (Figure 2(b)) , the inhibition of the mitochondrial activity significantly reduced the difference compared with that of the baseline level (20–40 min) (Figure 1(b)) , and FCCP injection significantly increased the OCR in the control groups compared with that of the FOXC1-deficient groups (40–80 min) (Figure 2(b)). However, when the mitochondrial function was totally disrupted by adding antimycin A and rotenone, which are inhibitors of mitochondrial complexes, OCR had no differences (80–100 min) (Figure 2(b)). These results indicated that the loss of FOXC1 enhanced aerobic glycolysis because of the impairment of mitochondrial functions in FOXC1-deficient cells.

Cancer cells undergo aerobic glycolysis and consume a high amount of glucose for their growth. Enhanced aerobic glycolysis was coupled by the increased concentration of extracellular acetic acids; thus, we also checked the ECAR levels. In Figure 1(c), at the baseline level (10–30 min), ECAR did not differ between the control and FOXC1-deficient groups. However, when glucose was added, the ECAR of the control group was significantly higher than that of the FOXC1-deficient groups. The ECAR of the control groups increased more significantly when the mitochondrial activity was inhibited by oligomycin, which is an ATP synthase inhibitor (60–100 min) (Figure 2(c)). 2-DG is a glucose molecule that could be uptaken by glucose transporters of cells, but they could not further undergo glycolysis. Thus, they would not form acetic acids. In Figure 1(c) (100–120 min), when 2-DG was added, ECAR had no difference. Moreover, the lactic acid content was significantly reduced when FOXC1 was downregulated (Figure 1(d)). All these results further confirmed that aerobic glycolysis was reduced when FOXC1 was downregulated in BLCA cells (253J and 5627 cell lines).

c-Myc is a downstream target of FOXC1 in several cancer types and is important for tumor progression. To explore whether the expression of c-Myc is affected in FOXC1-downregulated cell lines, we checked the expression of c-Myc by using a western blot and found that it was reduced significantly when theFOXC1 expression was downregulated (Supplementary Figure 2(a)). Therefore, c-Myc might be a downstream effector of FOXC1-mediated observations.

We explored how the loss of FOXC1 would affect tumor growth *in vivo* and injected FOXC1-deficient cells into nude mice to establish xenograft models. Tumor volumes were measured every 3 days by using a caliper. In Figures 1(e) and 1(f), tumor size and weight were significantly reduced in FOXC1-deficient mice, indicating that the loss of FOXC1 *in vivo* inhibits tumor growth. To further confirm whether proliferation was affected, we used Ki67 to stain the tumor sections and found few Ki67^+^-stained cells in FOXC1-deficient tumors, indicating that the loss of FOXC1 inhibits cell proliferation in these xenograft tumor models (Supplementary Figure 1(b)). Similar to *in vitro* studies, our research showed that the c-Myc expression decreased in FOXC1-downregulated tumors (Supplementary Figure 1(d)), which further supported a possible role of c-Myc in FOXC1-mediated observations.

### 3.3. Overexpression of FOXC1 Promotes Cell Proliferation, Increases OCR and ECAR, and Significantly Upregulates c-Myc Expression, While the Inhibition of c-Myc Reverses the Effects Exerted by FOXC1

To further confirm whether the observed phenotypes were due to the specific effect of FOXC1 knockdown, we overexpressed FOXC1 in one cell line, namely, T24, which has a low FOXC1 expression level (Supplementary Figure 1(d)). We successfully established the stable cell line and observed that FOXC1 was upregulated for over 10-folds in this stable cell line (oeFOXC1) (Supplementary Figure 1(d)). Using the CCK-8 assay, we found that FOXC1 overexpression in T24 cells could significantly promote cell growth *in vitro* (Figure 2(a)). We further checked the ORC, ECAR, and lactic acid content and found that the OCR, ECAR, and lactic acid content significantly increased when FOXC1 was overexpressed (Figures 2(b)–2(d)). Therefore, FOXC1 promoted cell growth through enhanced aerobic glycolysis.

Consistent with the abovementioned results, c-Myc expression was upregulated in FOXC1-overexpressed T24 cells (Supplementary Figure 2(d)). Thus, we showed that FOXC1 regulated BLCA cell growth by regulating aerobic glycolysis and c-Myc expression.

To further explore the role of c-Myc in this process, we used a c-Myc inhibitor, 10058-F4, to inhibit c-Myc in the control or FOXC1-overexpressing cells. We found that the inhibition of c-Myc could reverse the effects exerted by the overexpressed FOXC1 (Figures 2(e)–2(h)). Accordingly, we showed that this decrease in cell growth was coupled by a decrease in OCR, ECAR, and lactic acid content (Figures 2(e)–2(h)). Therefore, the FOXC1 regulation of c-Myc could affect cell growth by influencing aerobic glycolysis.

### 3.4. Exosome-Mediated Transfer of miR-4792 Inhibits Cell Growth, Reduces Aerobic Glycolysis, and Downregulates FOXC1 and C-Myc Expression

Exosomes are extracellular vesicles released by cells upon the fusion of an intermediate endocytic compartment. Exosomes could be uptaken by other cells and thus regulate their cellular function. miR-4792 is abundant in hAMSCs, so we extracted exosomes by using hAMSCs through ultrahigh-speed centrifugation. The content and purity of exosomes were determined under a transmission electron microscope. In Supplementary Figure 3(a), we extracted exosomes with good quality. We also confirmed our extracted exosomes by using exosomal markers, namely, TSG101 and CD81, through western blot. In Supplementary Figure 3(b), both markers could be detected in our extracted exosomes.

To study how the exosome-mediated transfer of miR-4792 could affect the cell function of BLCA cells, we first need to confirm that exosomes could be uptaken by BLCA cells. To check this finding, we used PKH-67 as a fluorescence tracing marker. After the BLCA cells (253J and 5637) were exposed to the exosomes, we found that the exosomes could be endocytosed by the 253J and 5637 cells (Supplementary Figure 3(c)).

**Figure 3:**
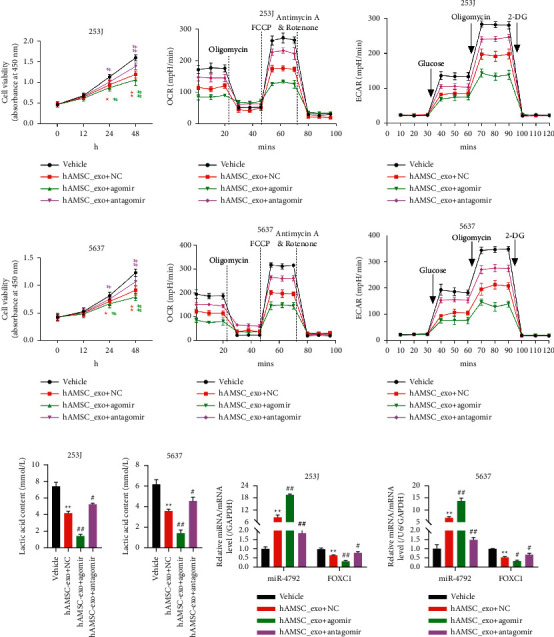
Exosome-mediated miR-4792 delivery significantly inhibited cell growth and aerobic glycolysis in 253J and 5637 cells. After miR-4792 inhibitor treatment (antagomir) and agomir mimic transfection of hAMSCs, exosomes were extracted from hAMSCs. (a) The CCK8 assay was used to measure the number of cells at 0, 12, 24, and 48 h ^*∗*^*p* < 0.05, ^*∗∗*^*p* < 0.01 vs. vehicle; #*p* < 0.05, ##*p* < 0.01 vs. hAMSC_exo + NC. (b, c) OCR and ECAR were measured using a Seahorse XF24 analyzer. (d) Lactic acid content was measured. (e) Expression levels of miR-4792 and FOXC1 were detected through qRT-PCR. ^*∗∗*^*p* < 0.01 vs. vehicle; #*p* < 0.05, ##*p* < 0.01 vs. hAMSC_exo + NC.

To further show how miR-4792 could exert its function, we used antagomir, which is a miR-4792 inhibitor, to inhibit the miR-4792 expression and agomir, which is a miR-4792 mimic, to upregulate the miR-4792 expression in hAMSCs. When exposed to the BLCA cells (253J and 5637), the exosomes contained a high level of miR-4792 that significantly inhibited cell growth compared with those of the control group (Figure 3(a)). However, when the miR-4792 level decreased, this inhibitory effect became less obvious than that of the control group (Figure 3(a)). Therefore, high levels of miR-4792 mediated by exosomes could suppress cell growth. In Figures 3(b)–3(d), the effects of the inhibited cell growth in the miR-4792 upregulation groups were accompanied by compromised aerobic glycolysis, as indicated by OCR, ECAR, and lactic acid content. qRT-PCR confirmed the expected miR-4792 levels in each of these groups (Figure 3(e)). These findings supported the observations exerted by the effect of miR-4792, but no other possible substances were found in the exosomes. A western blot further validated that high miR-4792 could inhibit the expression of FOXC1 and c-Myc (Supplementary Figure 3(d)). Therefore, miR-4792 could affect cell growth by regulating the expression of FOXC1 and c-Myc.

### 3.5. Forced Expression of FOXC1 Could Reverse the Effects Exerted by miR-4792

**Figure 4:**
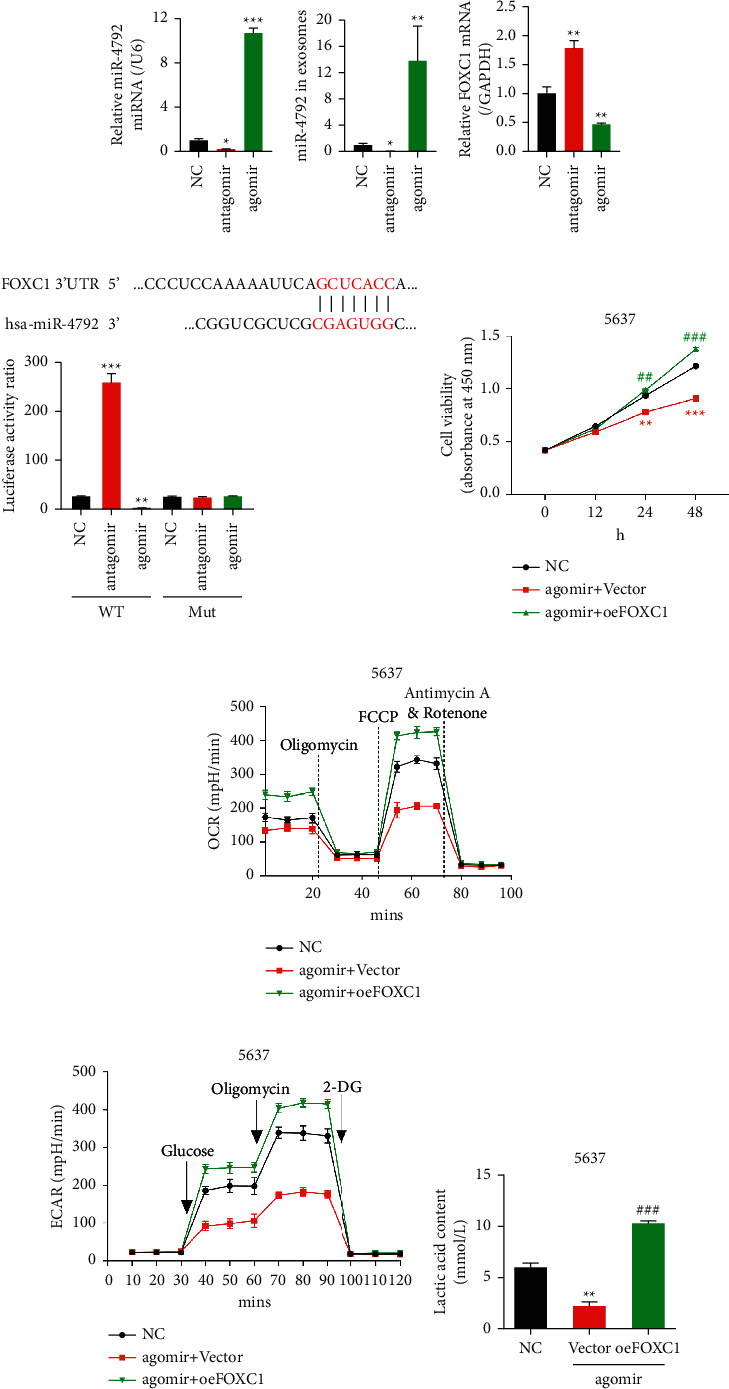
miR-4792 regulation of cell growth and aerobic glycolysis in BLCA cells is probably mediated by modulating the FOXC1 expression. (a–c) miR-4792 inhibitor treatment (antagomir) and agomir mimic transfection of hAMSCs were carried out on 5637 cells. (a, b) miR-4792 levels in hAMSCs. (c) mRNA level of FOXC1 in hAMSCs. (d) Detection of the luciferase activity ratio. ^*∗*^*p* < 0.05, ^*∗∗*^*p* < 0.01, ^*∗∗∗*^*p* < 0.001 vs. NC. (e–g) 5637 cells were untreated or transfected with agomir and empty vector or with agomir and FOXC1 plasmids (oxFOXC1). (e) The CCK8 assay was used to measure the number of cells at 0, 12, 24, and 48 h ^*∗∗*^*p* < 0.01, ^*∗∗∗*^*p* < 0.001 vs. NC; ##*p* < 0.01, ###*p* < 0.001 vs. agomir + vector. (f, g) OCR and ECAR were measured using a Seahorse XF24 analyzer. (h) Lactic acid content was measured. ^*∗∗*^*p* < 0.01 vs. NC; ###*p* < 0.001 vs. agomir + vector.

To further validate whether miR-4792 affected cell growth via FOXC1, we overexpressed FOXC1 in 5637 cells with high miR-4792 levels (agomir-treated group; Supplementary Figure 4(a)). The application of antagomir significantly decreased the miR-4792 level in 5637 cells and derived exosomes, whereas the application of agomir elicited reversed effects (Figures 4(a) and 4(b)). The FOXC1 expression was significantly promoted by antagomir and decreased by agomir (Figure 4(c)). The dual-luciferase assay revealed that antagomir and agomir could significantly enhance and reduce the luciferase activity in the cells transfected with wild-type FOXC1 vectors, respectively. Conversely, these effects were not detected in the cells transfected with mutant FOXC1 vectors (Figure 4(d)). Therefore, miR-4792 could bind to the 3ʹUTR of FOXC1 and decrease its expression. In Figure 4(e), high miR-4792 expression (agomir + vector group) could significantly inhibit cell growth; however, this effect could be reversed by the forced expression of FOXC1 (agomir + oeFOXC1), indicating that the miR-4792 function through the regulation of FOXC1 expression. This forced expression of FOXC1 also reversed the effects of miR-4792 on aerobic glycolysis, as shown by the increased OCR, ECAR, and lactic acid content (Figures 4(f)–4(h)). The forced expression of FOXC1 also reversed the protein expression level of c-Myc (Supplementary Figure 4(b)). Therefore, miR-4792 exerted its function by regulating the expression of FOXC1,c-Myc, and aerobic glycolysis.

### 3.6. miR-4792 Is Less Expressed, While FOXC1 Is Highly Expressed in Human Bladder Tumors

**Figure 5:**
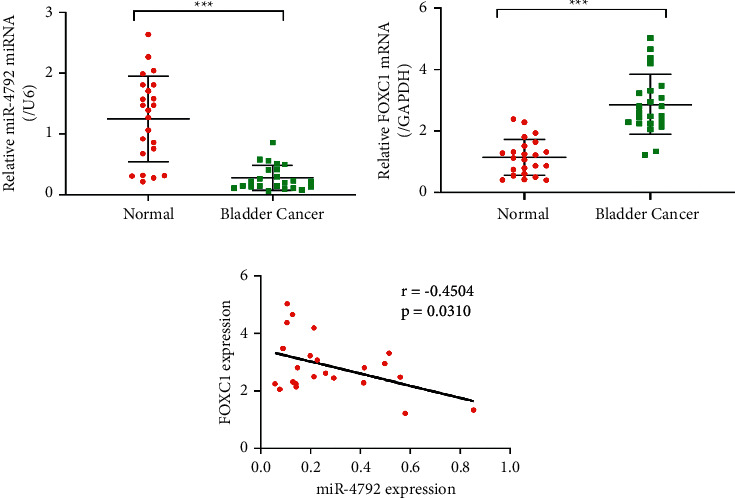
miR-4792 was poorly expressed in bladder tumor tissues, whereas FOXC1 was highly expressed compared with that of the paired adjacent normal tissues. Twenty-three pairs of tumor and normal adjacent tissues were collected. (a, b) mRNA expression levels of miR-4792 and FOXC1 were measured through qRT-PCR. (c) The Pearson correlation analysis between the expression levels of miR-4792 and FOXC1. ^*∗∗∗*^*p* < 0.001 vs. normal.

To confirm whether our observations in bladder cell lines and tumor models were applicable to patients with BLCA, we collected 23 BLCA tissues paired with adjacent normal tissues. Using qRT-PCR quantification, we showed that the miR-4792 expression was significantly lower in BLCA tissues than in adjacent normal tissues (Figure 5(a)). By contrast, FOXC1 was significantly higher in the former than in the latter (Figure 5(b)). The Pearson correlation analysis showed that the expression levels of miR-4792 and FOXC1 were significantly negatively correlated (Figure 5(c), *r* = −0.4504, *p* = 0.0310). These results showed that miR-4792 might have an important role in BLCA development through the regulation of the FOXC1 expression in these patients.

## 4. Discussion

Exosomes are regulators in intercellular communications and implicated in the development of cancers. They are abundant in proteins and RNAs, such as miRNAs, which allow the distant delivery of miRNAs. Recent studies have suggested that exosomal miRNAs can contribute to cancer development and progression [22]. miR-4792 is a tumor suppressor downregulated in several cancer types [16–19]. In non-small-cell lung cancer, miR-4792 can inhibit the proliferation and invasion of tumor cells [23]. Similarly, miR-4792 could decrease the expression of kindlin-3 to suppress the proliferation and invasion of acute myeloid leukemia cells and promote apoptosis [24]. In our study, we demonstrated that exosomal miR-4792 could be uptaken by BLCA cells. The high miR-4792 expression could inhibit the proliferation of BLCA cells and reduce aerobic glycolysis. Therefore, miR-4792 is a potential target for the treatment of BLCA.

Furthermore, the underlying mechanism of miR-4792 in regulating BLCA progression is explored. The loss of FOX protein plays an important role in tumorigenesis [25]. A previous study showed that miR-4792 can inhibit epithelial-mesenchymal transition and invasion in nasopharyngeal carcinoma by targeting FOXC1 [16]. Therefore, FOXC1 might be downstream of miR-4792 in bladder cancer. FOXC1 is associated with many biological processes, including development, cell differentiation, and apoptosis [5]. Studies have found that the upregulation of FOXC1 is related to the poor prognosis of a variety of human cancers, including acute myeloid leukemia [26], basal-like breast cancer [27], hepatocellular carcinoma [28], pancreatic duct adenocarcinoma [29], and gastric cancer [30]. Kumar et al. [31] revealed that the downregulation of FOXC1 can resensitize breast cancer cells to chemotherapeutic drugs, including doxorubicin and paclitaxel. FOXC1 can also promote the proliferation of gastric cancer cells by activating the Wnt signaling pathway [32]. However, the role of FOXC1 in BLCA remains unclear. Herein, our study first showed that FOXC1 was highly expressed in BLCA. The inhibition of FOXC1 significantly suppressed the proliferation and aerobic glycolysis of BLCA cells, whereas its overexpression exerted the opposite effects. Consistently, in vivo data also showed that the knockdown of FOXC1 significantly inhibited tumor growth. In addition, the luciferase reporter gene revealed that miR-4792 negatively regulated the promoter activity of FOXC1 3ʹUTR. Clinical analysis revealed that miR-4792 was weakly expressed in the bladder, whereas FOXC1 was highly expressed. miR-4792 was negatively correlated with FOXC1. hAMSC-exo significantly suppressed the proliferation and aerobic glycolysis of BLCA cells, which were enhanced by miR-4792 agomir and weakened by miR-4792 antagomir. Therefore, our study revealed a novel miR-4792/FOXC1 signaling pathway in BLCA pathogenesis.

The Warburg effect is a distinct form of cellular metabolism to increase the uptake of glucose and the production of lactate, which is commonly detected in cancer cells to promote their proliferation [33]. FOXC1 promotes the proliferation of colorectal cancer cells by inducing Warburg effects [34]. However, the role of FOXC1 in the Warburg effects of BLCA remains unclear. Our study revealed that aerobic glycolysis was significantly decreased by the knockdown of FOXC1. Furthermore, the elevation of miR-4792 could significantly reduce aerobic glycolysis, which was reversed by the FOXC1 overexpression. A previous study indicated that FOXC1 can promote the c-Myc expression in breast cancer [35], indicating the involvement of c-Myc underlying the oncogenic role of FOXC1. Consistent with previous findings, our data showed that the c-Myc expression levels *in vitro* and *in vivo* were reduced by FOXC1 knockdown, and the inhibition of c-Myc could reverse FOXC1-mediated effects. Therefore, our study indicated that miR-4792/FOXC1/c-Myc could promote the proliferation of BLCA cells by enhancing Warburg effects.

Although our study revealed a new role of miR-4792 and FOXC1 in BLCA, the exact regulatory mechanisms, such as how miR-4792 affects the expression of FOXC1, are unclear. We examined c-Myc but did not check other downstream regulators, such as matrix metalloprotease 7 [36], which might also affect BLCA development.

In summary, we showed a signaling axis where the exosome-mediated delivery of miR-4792 could regulate the expression of FOXC1 and c-Myc, which could further affect aerobic glycolysis and BLCA cell growth *in vitro* and *in vivo*. miR-4792 and FOXC1 might be used as potential drug targets for BLCA treatment.

## Data Availability

The datasets used and/or analyzed during the current study are available from the corresponding author on reasonable request.

## Supplementary Materials

Supplementary Materials Supplementary Figure 1: FOXC1 was highly expressed in bladder cancer cell lines, and stable cells deficient of or overexpressing FOXC1 have been established. Supplementary Figure 2: expression of FOXC1 and c-Myc in 253J and 5637 cells. Supplementary Figure 3: isolation and identification of exosomes from human adipose-derived mesenchymal stem cells (hAMSCs). Supplementary Figure 4: expression of FOXC1 and c-Myc in 5637 cell mutants. .
