# Supplementary figures and images for: Exosome-Mediated miR-4792 Transfer Promotes Bladder Cancer Cell Proliferation via Enhanced FOXC1/c-Myc Signaling and Warburg Effect
Source: J Oncol. 2022 Jan 19;2022:5680353. doi: 10.1155/2022/5680353 (PMC8791735; doi:10.1155/2022/5680353)

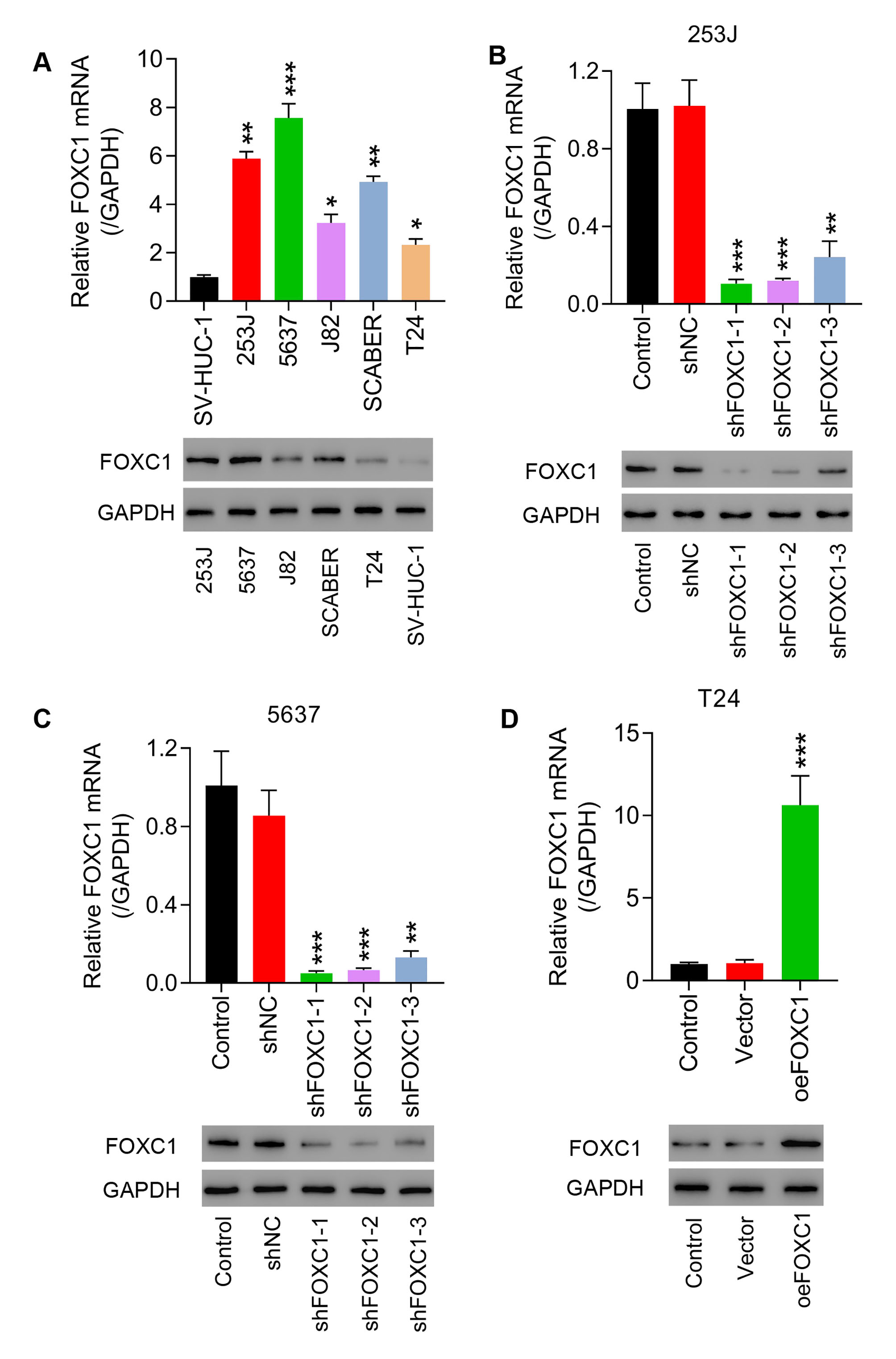

Supplement: Supplementary Materials — Supplementary Figure 1: FOXC1 was highly expressed in bladder cancer cell lines, and stable cells deficient of or overexpressing FOXC1 have been established. Supplementary Figure 2: expression of FOXC1 and c-Myc in 253J and 5637 cells. Supplementary Figure 3: isolation and identification of exosomes from human adipose-derived mesenchymal stem cells (hAMSCs). Supplementary Figure 4: expression of FOXC1 and c-Myc in 5637 cell mutants. . [file 5680353.f1.zip › 5680353.f1/Supplementary Figure 1.jpg]

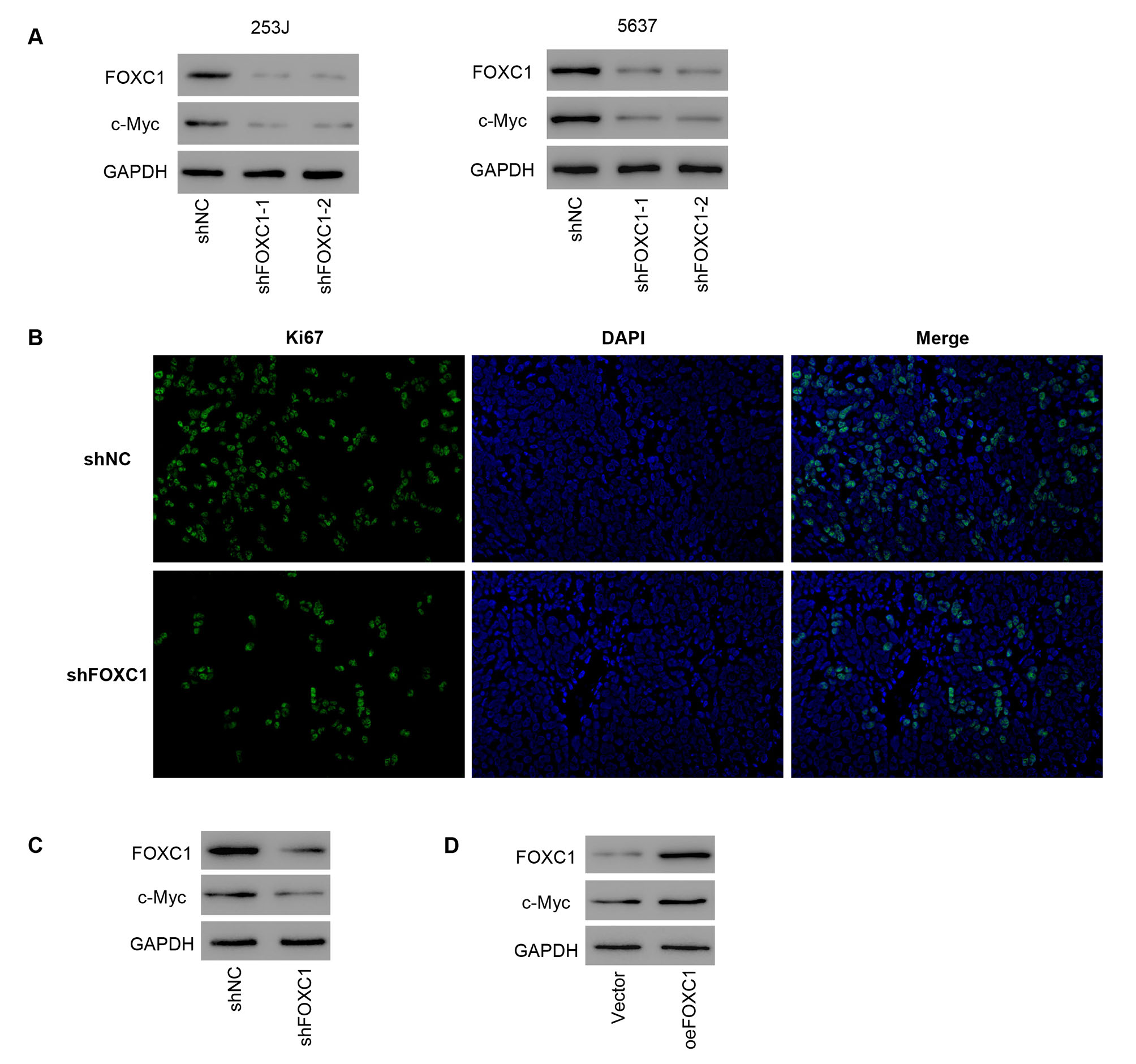

Supplement: Supplementary Materials — Supplementary Figure 1: FOXC1 was highly expressed in bladder cancer cell lines, and stable cells deficient of or overexpressing FOXC1 have been established. Supplementary Figure 2: expression of FOXC1 and c-Myc in 253J and 5637 cells. Supplementary Figure 3: isolation and identification of exosomes from human adipose-derived mesenchymal stem cells (hAMSCs). Supplementary Figure 4: expression of FOXC1 and c-Myc in 5637 cell mutants. . [file 5680353.f1.zip › 5680353.f1/Supplementary Figure 2.jpg]

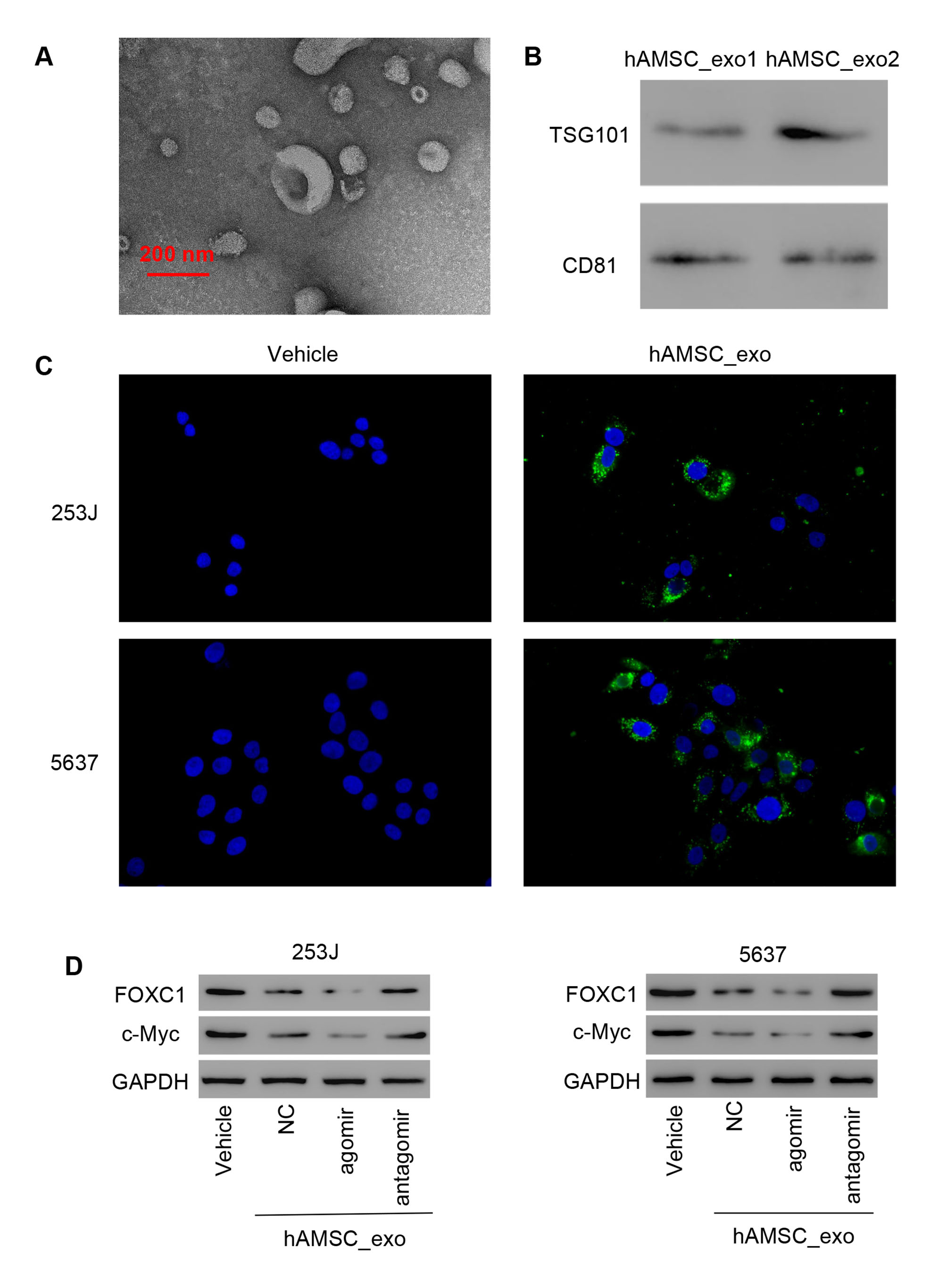

Supplement: Supplementary Materials — Supplementary Figure 1: FOXC1 was highly expressed in bladder cancer cell lines, and stable cells deficient of or overexpressing FOXC1 have been established. Supplementary Figure 2: expression of FOXC1 and c-Myc in 253J and 5637 cells. Supplementary Figure 3: isolation and identification of exosomes from human adipose-derived mesenchymal stem cells (hAMSCs). Supplementary Figure 4: expression of FOXC1 and c-Myc in 5637 cell mutants. . [file 5680353.f1.zip › 5680353.f1/Supplementary Figure 3.jpg]

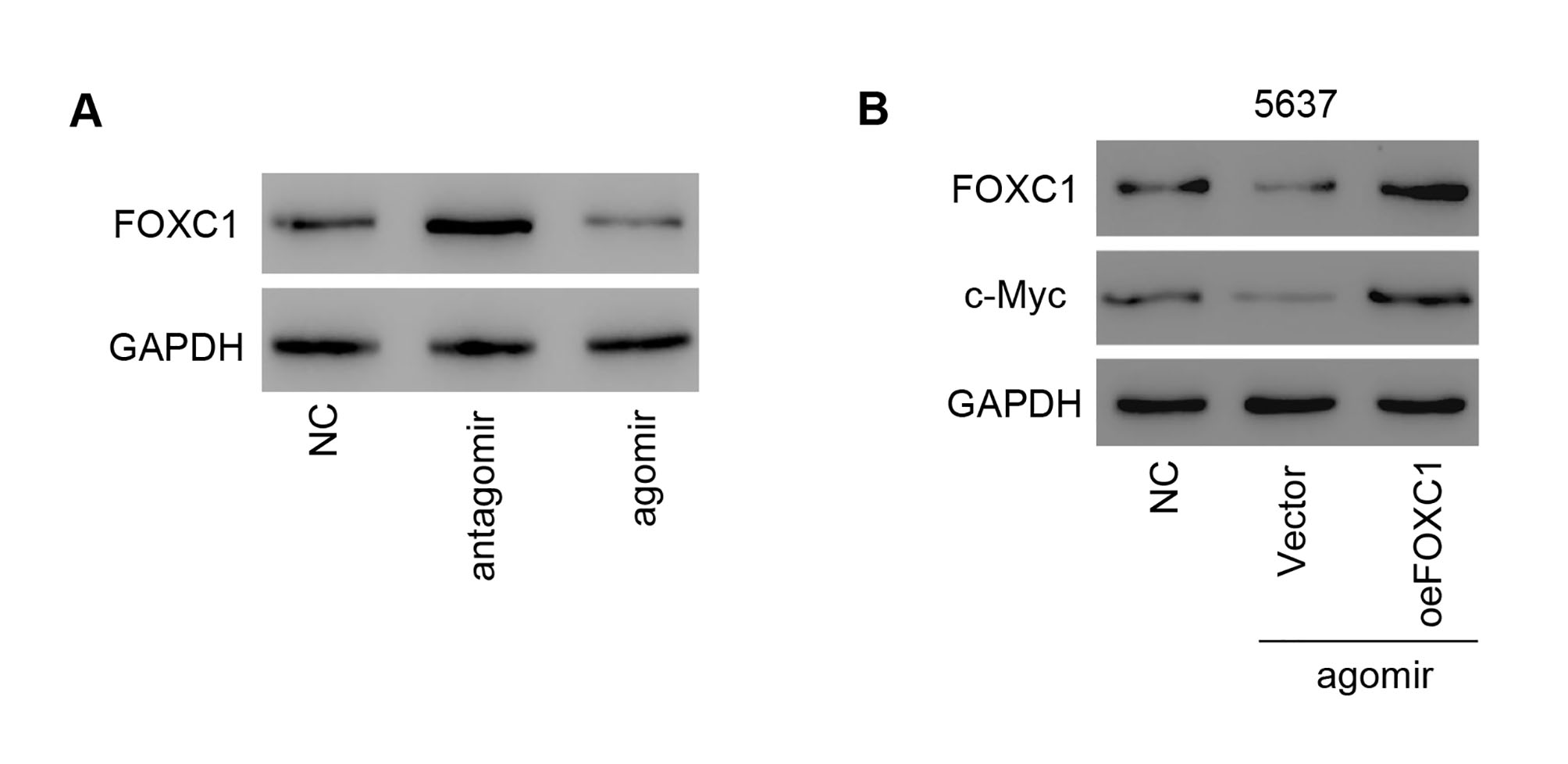

Supplement: Supplementary Materials — Supplementary Figure 1: FOXC1 was highly expressed in bladder cancer cell lines, and stable cells deficient of or overexpressing FOXC1 have been established. Supplementary Figure 2: expression of FOXC1 and c-Myc in 253J and 5637 cells. Supplementary Figure 3: isolation and identification of exosomes from human adipose-derived mesenchymal stem cells (hAMSCs). Supplementary Figure 4: expression of FOXC1 and c-Myc in 5637 cell mutants. . [file 5680353.f1.zip › 5680353.f1/Supplementary Figure 4.jpg]
